# Prevalence of Middle Mesial Canal in Mandibular Molars of a Saudi Subpopulation: A Prospective Cone-Beam Computed Tomography Study

**DOI:** 10.7759/cureus.47554

**Published:** 2023-10-24

**Authors:** Abdullah S Alsanah, Faris A Alqahtani, Dhafer M Alshehri, Abdullah M Alqahtani, Abdulaziz S AbuMelha

**Affiliations:** 1 Dentistry, King Khalid University, Abha, SAU; 2 Restorative Dental Sciences, King Khalid University College of Dentistry, Abha, SAU

**Keywords:** cone-beam computed tomography, endodontics, cbct, saudi arabia, mandibular molar, middle mesial canal

## Abstract

Background: Root canal treatment is compromised when one or more root canals are left unmanaged, especially in the case of multirooted teeth. Cone-beam computed tomography (CBCT) has the advantage of clearly recognizing the anatomical structures without any blurring and superimposition. There are few studies that assess the middle mesial canal (MMC) prevalence in the Saudi population using CBCT imaging. Thus, the present study was conducted to assess the MMC prevalence in the first and second mandibular molars in the Saudi population using CBCT imaging.

Materials and methods: Data from 616 patients and 1014 teeth were assessed. The mesial roots of the mandibular first and second molars were examined using CBCT to assess the presence of the MMC in both axial and coronal sections. The potential correlation between the prevalence of MMC was assessed with gender and age. The data collected were subjected to statistical analysis using IBM SPSS version 20.0 software (IBM Corp., Armonk, NY).

Results: The mean age was 34.39 ± 12.12 years, showing male predominance. A total of 2.6% and 0.2% cases of MMC were found in the first and second molars, respectively. A significant difference (p-value < 0.05) was observed among both genders in relation to age groups and the presence of MMC. MMC in the first molar was seen mainly in patients aged <20 years and only one case was reported with MMC in the second molar among patients aged 41-60 years.

Conclusion: A higher incidence of MMC was found in the first than the second mandibular molar. For accessing the MMC, the patient's age, high magnification, and troughing are some influential factors. In the future, clinical studies with long-term follow-ups are required to assess the influence of biomechanical preparation of MMC on the result of nonsurgical endodontic management in mandibular first as well as second molars.

## Introduction

Root canal therapy is aimed at removing various irritants (such as microorganisms and their byproducts and necrotic pulp tissue) from the root canal system. For successful endodontic therapy, it is important to have a detailed knowledge of the morphology and configuration of root canals of the tooth [1]. Disparity in roots and their morphology, such as the occurrence of fins, delta, loops, multiple orifices, and accessory canals, are common. Root canal treatment is compromised when one or more root canals are left unmanaged, especially in the case of multirooted teeth [2]. The most frequently endodontically treated tooth is the mandibular molar. It is a bi-rooted tooth, usually having one or two canals in the distal root and two root canals in the mesial root [3]. Various studies have revealed different variations in the root canal anatomy of mandibular molars, which are being determined by genetics and race. Variations observed were C-shaped anatomy of the roots, an isthmus between the mesiobuccal (MB) and mesiolingual (ML) canals, a separate distolingual root, and a third canal in the mesial root known as the middle mesial canal (MMC) [4].

The rate of prevalence of the MMC ranges from 0.2% to 50% [5]. Pomeranz et al. [6] categorized MMC into three classes: (1) canal starts from a different orifice, which continues independently without having interaction with ML or MB canals till the apex; (2) canal shows fusion and continues with either ML or MB canal showing confluence; and (3) canal is joined with an isthmus to ML or MB canal along its path showing finning. The most prevalent MMC type is confluent, followed by fin, and independent [5-7].

Different methods are being used to evaluate root canal morphology, such as clearing and staining of root canals, clinical studies, transparent tooth specimens, microscopic teeth sections, conventional radiography, micro-CT, and cone-beam computed tomography (CBCT). Recently, CBCT has attained huge recognition because of the advantage of clearly recognizing the anatomical structures without any blurring and superimposition that is usually seen in conventional two-dimensional imaging like panoramic and periapical radiography.

There are few studies that assess the MMC prevalence in the Saudi population using CBCT imaging. Thus, the present study was conducted to assess the MMC prevalence in the first and second mandibular molars in the Saudi population using CBCT imaging.

## Materials and methods

This prospective study was conducted in the Department of Restorative Dental Sciences, King Khalid University Dental Clinics, Saudi Arabia, from 2017 to 2022. Before starting the study, ethical approval was taken from the Institutional Review Board, King Khalid University, Kingdom of Saudi Arabia (IRB/KKUCOD/ETH/2022-23/011). Patients were explained about the study, and written informed consent was taken. CBCT data of patients who underwent radiographic scanning were reviewed.

A total of 806 patients were observed. Among those patients, 616 patients and 1014 teeth were included based on the inclusion criteria: (1) patients aged 18-60 years; (2) Saudi nationality; and (3) presence of either first or second mandibular molars. The exclusion criteria were (1) non-Saudi patients, (2) age less than 18 years (as roots may not be completely formed) and above 60 years (as roots may become calcified), and (3) missing first and second mandibular molars.

The mesial roots of the mandibular first and second molars were examined using CBCT (KKGT-0060, KaVo Kerr, Biberach, Germany) by two trained clinicians to assess the presence of the MMC. The MMC was recorded when clearly seen in both axial and coronal sections. The CBCT data volumes with different voxel sizes might have led to the inability to detect some MMC, as a narrow MMC might be missed when a larger voxel size is used. The potential correlation between the prevalence of the MMC was assessed and correlated with the gender and age of patients. The data collection was subjected to statistical analysis using IBM SPSS version 20.0 software (IBM Corp., Armonk, NY).

## Results

The maximum (62.99%) cases were aged 20-40 years, with the mean age being 34.39 ± 12.12 years, showing male predominance. It was observed that in 2.6% and 0.2% of cases, the MMC was found in the first and second molars, respectively (Table 1).

**Table 1:** Demographic data MMC: middle mesial canal.

Parameters		Frequency (n)	Percentage (%)
Age groups (years)	<20	49	7.954
	20-40	388	62.99
	41-60	154	25
	>60	25	4.06
Gender	Female	285	46.3
	Male	331	53.7
MMC 1st molar	Molar agenesis	145	23.5
	Absent	455	73.9
	Present	16	2.6
MMC 2nd molar	Molar agenesis	73	11.9
	Absent	542	88.0
	Present	1	0.2
Total		616	100.0
Mean age		34.3912	12.12152

**Figure 1:**
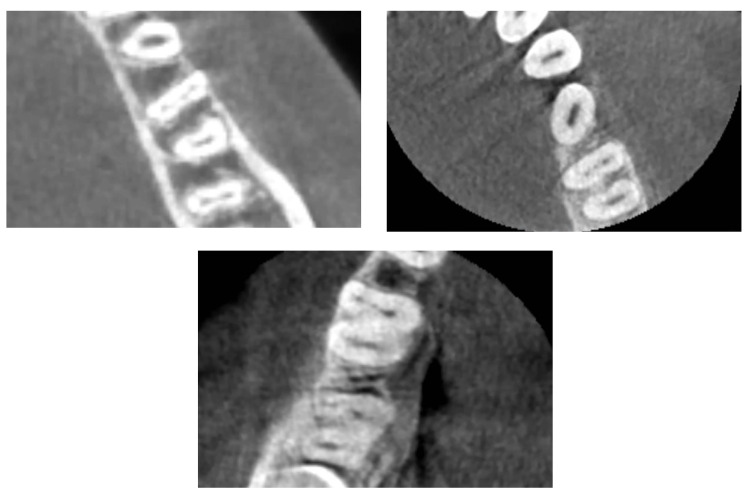
Mandibular first molars with middle mesial canals

Among males, more cases of MMC were observed in both the molars. It was found that a significant difference (p-value < 0.05) was observed among both genders in relation to age groups and the presence of MMC (Table 2 and Figure 1).

**Table 2:** Correlation between both genders in relation to various parameters * P-value < 0.05 is significant. MMC: middle mesial canal.

Parameters		Male (n = 331)		Female (n = 285)	
		Frequency (n)	Percentage (%)	Frequency (n)	Percentage (%)
Age groups	<20	24	7.25	25.0	8.77
	20-40	194	58.61	194.0	68.07
	41-60	98	29.61	56.0	19.65
	>60	15	4.53	10.0	3.51
Chi-square		1.127			
p-value		0.051*			
MMC 1st molar	Molar agenesis	85	25.7	60	21.1
	Absent	235	71.0	220	77.2
	Present	11	3.3	5	1.8
Chi-square		2.019			
p-value		0.032*			
MMC 2nd molar	Molar agenesis	32	9.7	41	14.4
	Absent	298	90.0	244	85.6
	Present	1	0.3	0	0
Chi-square		2.289			
p-value		0.043*			

A greater number of males was seen in age groups above 40 years of age. MMC in the first molar was seen mainly in patients aged <20 years, followed by 41-60 years and 20-40 years of age. Only one case was reported with MMC in the second molar among patients aged 41-60 years (Table 3).

**Table 3:** Correlation between age groups in relation to various parameters * P-value < 0.05 is significant. MMC: middle mesial canal.

Parameters		<20		20-40		41-60		>60	
		n	%	n	%	n	%	n	%
Mean age (mean ± SD)		18.20	0.41	29.13	5.44	47.95	5.28	64.24	4.21
Gender	Female	25	51.0	194	50.0	56	36.4	10	40.0
	Male	24	49.0	194	50.0	98	63.6	15	60.0
Chi-square		1.718							
p-value		0.042*							
MMC 1st molar	Molar agenesis	10	20.4	86	22.2	43	27.9	6	24.0
	Absent	37	75.5	293	75.5	106	68.8	19	76.0
	Present	2	4.1	9	2.3	5	3.2	0	0
Chi-square		2.167							
p-value		0.018*							
MMC 2nd molar	Molar agenesis	3	6.1	43	11.1	23	14.9	4	16.0
	Absent	46	93.9	345	88.9	130	84.4	21	84.0
	Present	0	0	0	0	1	0.6	0	0
Chi-square		2.007							
p-value		0.002*							

## Discussion

In clinical practice, mandibular molars are the most commonly encountered teeth requiring endodontic treatment, as they are the most vulnerable to the development of dental caries, leading to endodontic treatment [8]. The successful endodontic treatment is based on meticulous instrumentation and thorough irrigation, followed by obturation. Disparity in the morphology of root canals can be a challenge to clinicians, mainly deviations in the MMC of roots of first and second mandibular molars, as it is rare in occurrence. It needs an additional endeavor for identifying and treating MMC. Various interventions are being utilized for evaluating the incidence of MMC, such as clinical studies, dental operating microscopes, intraoral radiography, CBCT, and micro-CT [9]. In recent years, the utilization of CBCT imaging has become famous among endodontists, as it provides images of oral tissues without blurring or superimposition, which is commonly observed in panoramic and intraoral radiographs. Thus, the present study was conducted on the first and second mandibular molars using CBCT imaging [10].

In our study, the maximum cases were aged 20-40 years, with the mean age being 34.39 ± 12.12 years, showing male predominance. Similar to our study, Peiris et al. [11] suggested that the rate of incidence of MMC is higher among patients aged 30-40 years old, coinciding with the time of completion of differentiation of the root canal. They stated that this is because of changes in the configuration of the root canal and maturation after the root development completion and apex closure. A constant secondary dentin deposition occurs within the root canals that causes a more complicated configuration of the root canal with the chances of formation of a third root canal in the mesial root of the first and second mandibular molars [12].

In our study, it was observed that in 2.6% and 0.2% of cases, MMC was found in the first and second molars, respectively. Aldosimani et al. [1] observed that MMC was found in 1.3% of cases in first molars and in 0.4% of cases of second molars. Kim et al. [13] observed a lower prevalence rate of 0.35% of MMC in the first mandibular molar. Similar to our study, Wang et al. [14] found a prevalence rate of 2.7% in the first mandibular molar. Srivastava et al. [15] observed a considerably higher rate of prevalence of MMC as compared to the results of our study with a prevalence of 18.2%.

We also found that among males, more cases of MMC were observed in both the molars. It was found that a significant difference (p-value < 0.05) was observed among both genders in relation to age groups and the presence of MMC. A greater number of males was seen in age groups above 40 years of age. MMC in the first molar was seen mainly in patients aged <20 years, followed by 41-60 years and 20-40 years of age. Only one case was reported with MMC in the second molar among patients aged 41-60 years of age. Kuzekanani et al. [3] observed that the incidence of MMC was higher among females than males. The variability observed in results can be due to various causes like study design, sample size, region, and population studied.

Increased knowledge of variations in morphological patterns of molars can enhance the success rate for both surgical and non-surgical root canal treatment, thus avoiding treatment failure. In vivo CBCT analysis is a clinically effective and non-invasive tool that determines the morphology of the root and root canal [9].

The limitation of the current study is the CBCT data volumes with different voxel sizes might have led to the inability to detect some MMC, as a narrow MMC might be missed when a larger voxel size is used. The use of limited-field CBCT scans is recommended to make sure smaller voxel sizes are used.

## Conclusions

This study revealed a higher incidence of MMC in the first than the second mandibular molar. The identification and biomechanical cleaning of MMC during surgical or nonsurgical root canal treatment is crucial. For accessing the MMC, the age of the patient, high magnification, and troughing are some influential factors. In the future, clinical studies with long-term follow-ups are required to assess the influence of biomechanical preparation of MMC on the result of nonsurgical endodontic management in mandibular first as well as second molars.
